# Sciatica Caused by Perineural Spread of Prostate Cancer

**DOI:** 10.7759/cureus.38057

**Published:** 2023-04-24

**Authors:** Shunsuke Katsumi, Shigeru Soshi, Takayoshi Kajiwara, Mitsuru Saito

**Affiliations:** 1 Department of Orthopedic Surgery, The Jikei University School of Medicine, Tokyo, JPN

**Keywords:** sciatica, prostate cancer, perineural spread, sciatic nerve, metastasis, spine surgery, degenerative lumbar disease

## Abstract

An 81-year-old man with a history of prostate cancer developed sciatica and underwent L4/5 laminectomy followed by L5/S1 transforaminal lumbar interbody fusion. Postoperatively, pain improved temporarily, then deteriorated. Tumor resection was performed after enhanced magnetic resonance imaging showed a mass distal to the left greater sciatic foramen. Histopathological examination showed the perineural spread of prostate cancer to the sciatic nerve. Developments in diagnostic imaging have revealed that prostate cancer can undergo perineural spread. Imaging studies are essential when sciatica is diagnosed in patients with a history of prostate cancer.

## Introduction

Prostate cancer is the most common tumor in elderly men [1]. Prostate cancer usually metastasizes to the bones and lymph nodes or invades directly into the surrounding tissue. However, prostate cancer can, on rare occasions, metastasize into the pelvic plexus or sciatic nerve. In such cases, cancer progression does not occur by hematogenous metastasis, lymphatic metastasis, or direct invasion but rather spreads via the perineurium [2]. This report describes a rare case of sciatica due to the perineural spread of prostate cancer to the pelvic plexus. The patient, in this case, underwent multiple operations because of the difficulty of differentiating the pathology from degenerative lumbar disease.

## Case presentation

**Figure 1:**
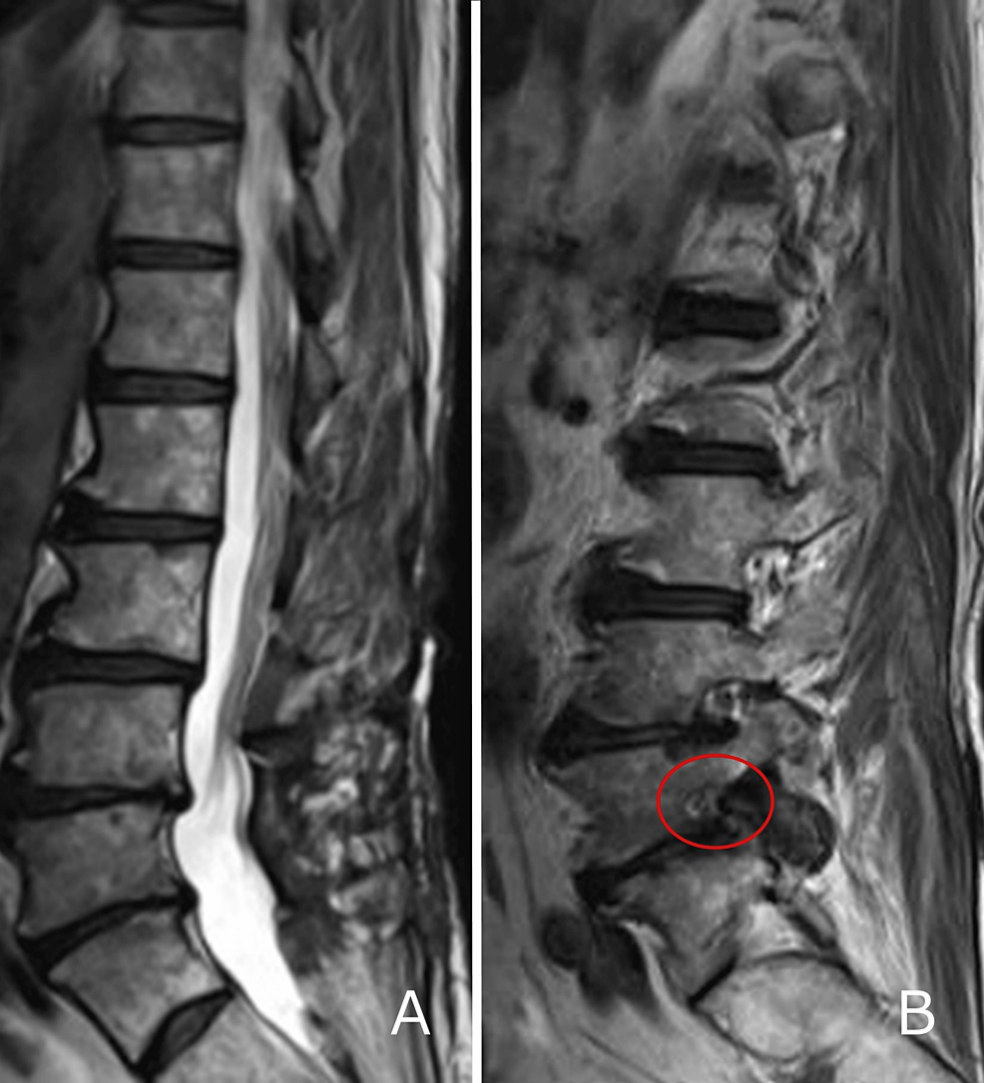
Preoperative T2-weighted magnetic resonance imaging (A) No stenosis of the spinal canal is evident. (B) Stenosis is apparent at the left L5/S1 foramen.

**Figure 2:**
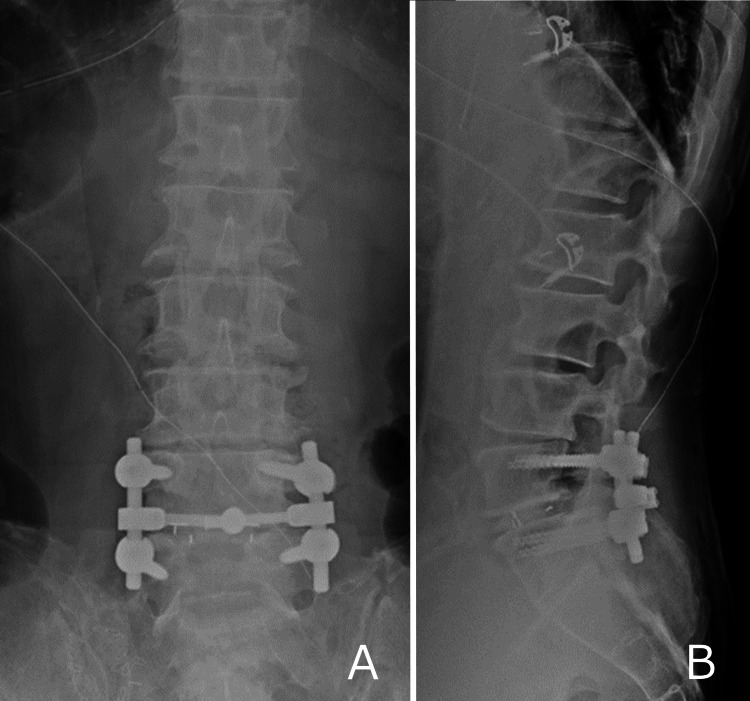
Postoperative X-ray images After L5/S1 transforaminal lumbar interbody fusion. (A) Anteroposterior view. (B) Lateral view.

An 81-year-old man had undergone total prostatectomy and endocrine therapy for prostate cancer 16 years earlier, with no recurrence observed until this presentation. The only other relevant medical history was hypertension. His chief complaint was left sciatica, and stenosis of the L4/5 spinal canal was diagnosed. Decompression surgery was then performed in another hospital. Left leg pain remained postoperatively, and the patient visited our hospital. Although the results of motor and sensory examinations were normal, he experienced difficulty walking due to intermittent claudication and severe pain in the left buttock and posterior thigh. Straight leg raising tests showed negative results bilaterally. Patellar and Achilles tendon reflexes were normal. Blood biochemical tests showed a mildly elevated prostate-specific antigen (PSA) level of 10.93 ng/ml but no other abnormalities. Magnetic resonance imaging (MRI) did not show any spinal canal stenosis, including at the previous surgery site at L4/5. However, foraminal stenosis was observed at the left L5/S1 level. A left L5 nerve root block was performed, confirming referred pain and providing pain relief (numerical rating score: from 10 before block to 5 after block). Based on these findings, we diagnosed L5/S1 foraminal stenosis and performed decompression surgery with transforaminal lumbar interbody fusion (Figures 1, 2).

**Figure 3:**
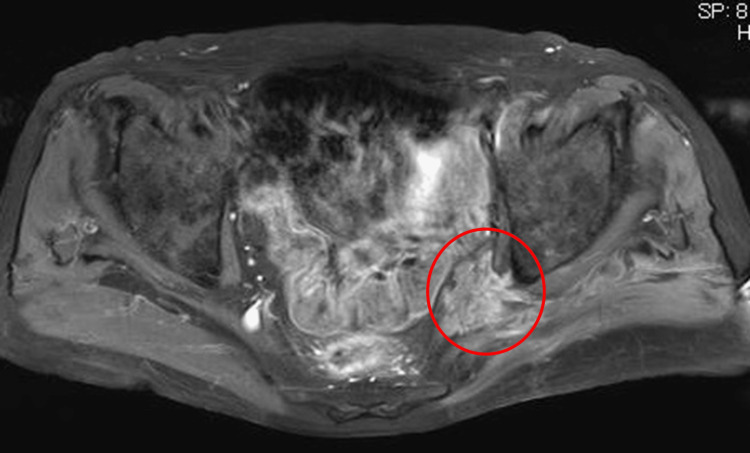
Pelvic enhanced magnetic resonance imaging Enhanced magnetic resonance imaging (MRI) shows a mass with indistinct borders and homogeneous contrast in the region of the left greater sciatic foramen.

Intraoperative findings showed severe stenosis at the L5/S1 intervertebral foramen but mild stenosis at the L5/S1 disc level. Postoperatively, left buttock pain improved temporarily before deteriorating within a few weeks. Tenderness was identified at the Valleix points in the sciatic notch and posterior thigh. The postoperative status of the lumbar spine was examined, with no obvious findings on MRI. A pathology other than a lumbar lesion was therefore suspected, and the pelvis was explored on MRI. Enhanced MRI revealed a mass showing homogeneous contrast with indistinct borders distal to the left greater sciatic foramen (Figure 3).

**Figure 4:**
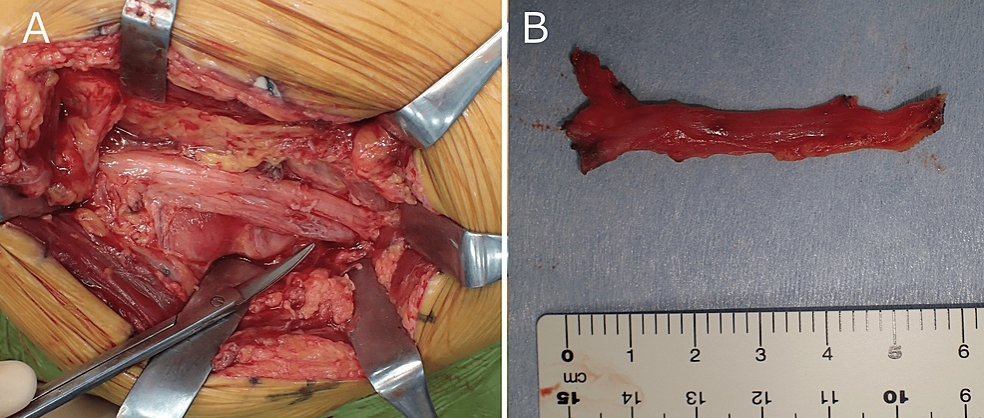
Intraoperative images (A) Intraoperative photograph. (B) A resected specimen of the perineural sciatic nerve at the site shows contrast enhancement on magnetic resonance imaging (MRI).

On suspicion that the sciatic nerve pain was attributable to a tumor, tissue around the sciatic nerve was resected along with part of the sciatic nerve. The pain improved immediately after this surgery (Figure 4).

**Figure 5:**
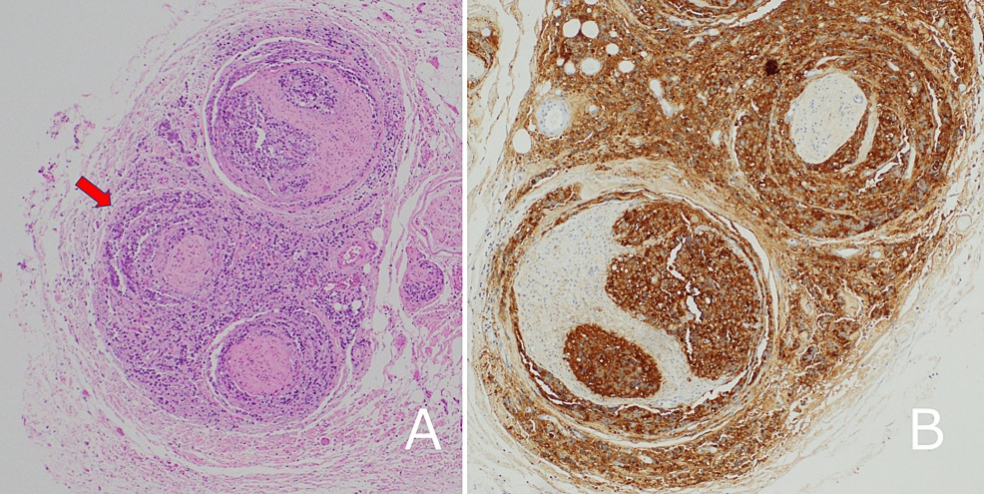
Histopathological images of the resected specimen. (A) The tumor is confined to the perineurium (arrow). Hematoxylin and eosin staining (HE); 40× magnification. (B) Positive immunohistochemical staining for prostate-specific antigen in tumor cells; 100× magnification.

Histopathological examination revealed that the tumor was concentrated around the perineurium, with some tumors also present in the endoneurium. Immunostaining showed positive results for PSA. On the other hand, no tumor was identified in the epineurium, leading to the diagnosis of the perineural spread of prostate cancer (Figure 5).

As of one year postoperatively, the patient had experienced no recurrence of pain.

## Discussion

In Japanese men, morbidity rates for prostate cancer exceeded those for gastric cancer in 2018 and seem likely to keep increasing [1]. In addition, because of the good five-year survival rate, the number of patients with prostate cancer is steadily increasing [3]. Moreover, considering that bone metastasis is very common among those patients, orthopedic treatment is quite important.

Most cases of sciatica are due to lumbar disease, such as spinal canal stenosis, disc herniation [4], or intraspinal tumors (schwannoma, meningioma) [5]. On the other hand, a few cases involve tumors in the retroperitoneal space (rectal cancer, gynecological and prostatic malignancies, urinary bladder carcinoma, retroperitoneal sarcoma), trauma, infection, gynecological disease, or pisiform muscle syndrome [6,7]. When performing spinal surgery, all of these possibilities must be considered, and a precise diagnosis must be made. Among women, endometriosis, ovarian cysts, and pregnancy can cause sciatica [8]. Osteoporotic vertebral fractures and fragility fractures of the sacrum can also cause sciatica. We should pay attention to occult fractures of the sacrum in elderly individuals [9] because an obvious history of trauma is often lacking. Infections such as spondylodiscitis and abscesses in the iliopsoas muscle, pelvic cavity, or gluteal muscle [10] should be suspected when fever or a worsening inflammatory response is present.

Prostate cancer is known to metastasize via the lymphatic system to pelvic or abdominal lymph nodes or hematogenous to the bone. Invasion of the lumbosacral plexus by prostate cancer is usually attributed to direct invasion of the plexus, either by the prostate cancer itself or by metastases adjacent to the plexus. In the present case, direct invasion of the epineurium, the outermost portion of the peripheral nerves, would have been suspected. However, in 2006, Ladha et al. reported the first case in which prostate cancer invasion was confined to the endoneurium of the lumbosacral plexus with no evidence of pelvic or extraprostatic invasion [2]. This suggested a different pattern of invasion from previous reports. In 2010, Hébert-Blouin et al. retrospectively evaluated MRI findings from patients diagnosed with lumbosacral plexus disorders due to prostate cancer and reported the histological findings after a biopsy of the sciatic nerve showed perineural spread from the pelvic plexus to the lumbosacral plexus and the sciatic or sacral spinal nerves [11]. Malignant melanoma of the head and neck has been reported to show retrograde spread along the cutaneous nerves and invasion of the central intracranial nerves [12]. We, therefore, supposed that retrograde perineural spread along the nerves innervating the prostate (S2-4) could occur in the same manner as a perineural spread. In 2013, Babu et al. reported that high-resolution MRI and positron emission tomography CT (PET-CT) with intrarectal coils are useful for diagnosing perineural spread [13]. In an MRI of the lumbar spine, fat-suppressed 3-dimensional coronal imaging might be useful to reveal the lesion not only in foraminal portions but also at extra-foraminal portions, although abnormal findings in those regions can be difficult to identify on axial images. We recommend both pelvic MRI and PET-CT for patients who have undergone previous treatment for prostate cancer, particularly in patients who show severe sciatica.

Similar to previous reports, the current case showed MRI findings consistent with enhancement along the sciatic nerve, pathological evidence of metastasis localized to the endoneurium, and sciatica. A literature search identified eight cases of perineural spread due to prostate cancer [14]. Among the patients, seven had unilateral sciatica, while one had bilateral sciatica. Additionally, six of the eight patients showed muscle weakness, and seven displayed sensory disturbances. Precise mechanisms underlying sciatic nerve pain due to the perineural spread of prostate cancer have taken a long time to clarify (meantime, 9.3 years; range, 3 months to 18 years). Compared to sciatica caused by lumbar degenerative disease, sciatic nerve pain due to the perineural spread of prostate cancer may be more severe. Although patients with lumbar degenerative disease and the current patient with prostate cancer presented with sciatica, it is difficult to distinguish between the two diseases based on the effect of nerve root blocks, as both types of pain can be reduced by such blocks. The most notable feature of the current case was that a long period elapsed between the diagnosis of prostate cancer and the onset of the first symptoms, with sciatic nerve pain occurring 15 years after the prostate cancer diagnosis. The treatment histories and disease durations of patients with perineural spread were diverse. Regardless of the treatment of prostate cancer, we believe that all prostate cancer patients who develop sciatica should have an MRI or PET-CT. Spine and orthopedic surgeons should be aware of the possibility of this condition and should not overlook it.

## Conclusions

We have described a case of sciatica caused by the perineural spread of prostate cancer. With the development of diagnostic imaging modalities such as MRI and PET-CT, perineural spread from prostate cancer has become evident as a possibility. We, as orthopedic surgeons, must keep this condition in mind and perform imaging studies when diagnosing sciatica in a patient with a history of prostate cancer.
